# Financial loss estimation of bovine fasciolosis in slaughtered cattle in South Africa

**DOI:** 10.1016/j.parepi.2017.10.001

**Published:** 2017-10-12

**Authors:** Ishmael Festus Jaja, Borden Mushonga, Ezekiel Green, Voster Muchenje

**Affiliations:** a Department of Livestock and Pasture Science, University of Fort Hare, Alice 5700, South Africa; b Department of Biomedical Sciences, School of Veterinary Medicine, Faculty of Agriculture and Natural Resources, University of Namibia, Namibia; c Department of Biochemistry and Microbiology, University of Fort Hare, Alice 5700, South Africa

**Keywords:** Abattoir, Cattle, Economic loss, Food safety, *Fasciola*, Liver condemnation, South Africa

## Abstract

South Africa's livestock population is rapidly evolving and consequently will require sustained epidemiological surveillance to detect and prevent diseases which contribute to a decrease in livestock productivity, public health risk and food wastage. *Fasciola* infection is one of the commonest diseases affecting livestock health and a significant portion of meat and offal's are declared unfit for human consumption. This study assessed the prevalence and monetary losses associated with *Fasciola* infection at three abattoirs in Eastern Cape Province. A retrospective data of all slaughtered cattle were obtained from Department of Rural Development and Agrarian Reform (DRDAR) from 2010–2012. A prospective abattoir survey was conducted between July 2013 and June 2014 to determine the prevalence and financial loss due to fasciolosis.

The highest prevalence was in December and January (23%) while the lowest prevalence was recorded in May and June (5%). Annual prevalence of *Fasciola* at abattoirs AB1, were (3.2%, 2.2% and 2.0%), AB2 (6.4%, 4.6% and 3.5%), AB3 (14.4%, 6.9% and 9.5%) for year 2010, 2011 and 2012 respectively. The total financial loss due to *Fasciola* infection during the active survey of the three abattoirs was ZAR 44, 930 (3456.2 USD). A breakdown of this figure shows that whole liver condemnation was ZAR 25, 230 (2, 357 USD), and partial liver condemnation was ZAR 19, 700 (1515.4 USD).

The present study reveals the economic loss due to liver condemnation from *Fasciola* infection and provides regional baseline information regarding the prevalence of *Fasciola* in cattle at three abattoirs.

## 1 Introduction

*Fasciola* is a trematode predominantly found in ruminants (cattle, buffalo, sheep, and goats), but can also infect humans (Ashrafi and Mas-Coma, 2014). The infection is cosmopolitan with *Fasciola hepatica* and *Fasciola gigantica* being the most prominent cause of fasciolosis. Despite evidence of lack of proper documentation of disease burden in livestock, in the tropics, *Fasciola* infection is regarded as the most important parasitic disease in ruminant with prevalence ranging from 25 to 100% (Toet et al., 2014, Torgerson and Macpherson, 2011, Tsegaye et al., 2011). Infection in livestock usually leads to reduced growth, poor production of meat and milk. Other complications of *Fasciola* infection are reduced fertility, abortion in late stages of pregnancy, anemia and mortality. In dairy cattle, reduction in milk yield, due to infection with Fasciola Spp. is between 3.8% to 15.2% while global production losses exceed US$3 billion/year (Bekele et al., 2010, Elliott et al., 2015, Jean-Richard et al., 2014, Martínez-Pérez et al., 2012, Terefe et al., 2012, Toet et al., 2014). The parasite activity damages the liver and leads it condemnation. When the trematode reaches the bile ducts and attains sexual maturity, some parasite eggs migrate to liver parenchyma causing severe eosinophilic and granulomatous inflammatory responses (Buffoni et al., 2010, Martínez-Pérez et al., 2012, Molina-Hernández et al., 2015, Zafra et al., 2010). Several studies have shown that it is at this stage that most pathological damage starts to occur. (Keyyu et al., 2006, Khan et al., 2010, Sánchez-Andrade et al., 2002, Tsotetsi and Mbati, 2003).

This infection is a major veterinary disease and has recently been shown as a significant public health problem (Ashrafi and Mas-Coma, 2014, Mas-Coma et al., 2005). Human disease has been reported in five continents and about 2.4 million person are infected in 61 countries and much more are at risk of the infection (Molina-Hernández et al., 2015, Torgerson and Macpherson, 2011). In South Africa, three cases had previously been in 1964, and two new cases recently reported in the Western Cape Province (Black et al., 2013).

There are other challenges to *Fasciola* infection including the costs of treatment in high prevalence and endemic areas, and the risk of drug residues in food animal, parasites resistant to the frontline drug (triclabendazole). In South Africa, the average cost of treatment of fasciolosis per animal per is ZAR 15–20. The burden of the disease and cost treatment harms sustainable livestock production (Quayle et al., 2010). Global warming also significantly favour the replication of *Galba truncatula* (intermediate host) (Buffoni et al., 2010, Molina-Hernández et al., 2015). *Galba truncatula* and *Radix natalensis* have been reported in South Africa, and *R. natalensis* is regarded as the most common intermediate host of *F. gigantica* in the country. *Galba truncatula,* is also a good aestivator contributing to its geographical spread even in unfavorable clime (De Kock and Wolmarans, 2011, Kock et al., 2003).

Food safety concerns are compelling reasons for meat inspection and condemnation of infected liver. In this regard, the abattoir play a crucial role not only in the detection and elimination of unhealthy meat from the food chain, but also a source of useful epidemiological data (Alton et al., 2012, Blagojevic and Antic, 2014, Phiri, 2006, Soji et al., 2015, Thomas-Bachli et al., 2012). Reports on economic losses due to condemnation of *Fasciola* infected liver in South Africa are scanty. Nonetheless, elsewhere in Africa, studies have shown condemnation rates of 8–57% (Alawa et al., 2011, Fekadu et al., 2012, Mellau et al., 2011, Mellau et al., 2010, Pfukenyi and Mukaratirwa, 2004, Phiri, 2006).

Therefore, this study aims to determine the prevalence of *Fasciola* and estimate financial loss associated with fasciolosis in slaughtered cattle in selected abattoirs in the Eastern Cape Province, South Africa.

## 2 Materials and methods

### 2.1 Ethical consideration

Ethical clearance number MUS071SJAJ01 was obtained from the University of Fort Hare research ethics committee before the commencement of field data collection. Official permission was obtained from the Department of Rural Development and Agrarian Reform (DRDAR) and participating abattoirs.

### 2.2 Description of study area, animal husbandry and selected abattoirs

**Fig. 1:**
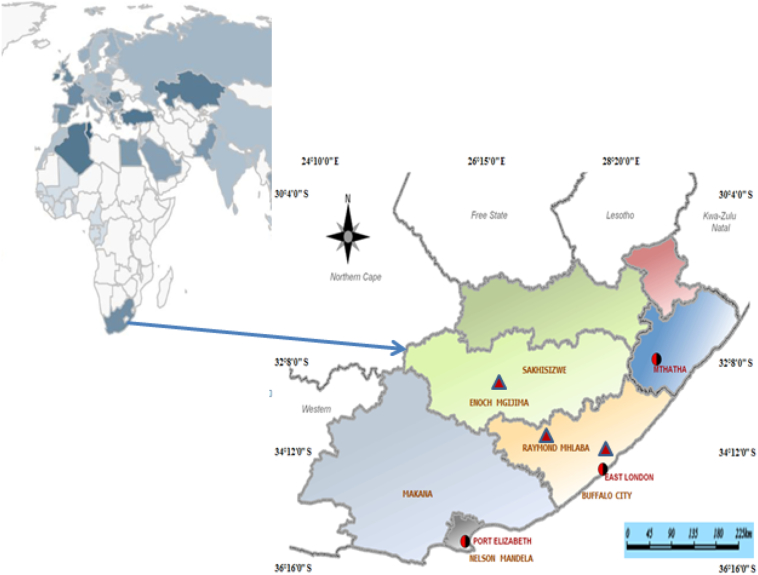
Map of Africa with a pointer to the Eastern Cape Province. Triangles show the location of abattoir.

Eastern Cape Province (ECP) is located at latitude 32° S and longitude 26° E in southeastern South Africa. It is the third most populated and rural Province with 63.4% of the population living in rural areas (Carabin et al., 2006). Cattle ownership and rearing are popular in the Province. Cattle population in South Africa is estimated to be above 14.1 million; about 3.2 million is in reared in the Eastern Cape representing about 22.6%. Semi-intensive breeding is widely applied by commercial farms whereas extensive management system is common among rural and smallholder farms. Three abattoirs (AB1, AB2, and AB3) were selected for this study (Fig. 1). AB1 and AB2 were high throughput abattoir located in East London and Queenstown respectively, while AB3 was a low throughput abattoir is located in Adelaide (Jaja et al., 2016). A low throughput abattoir slaughters between 3 and 21 animals per day while high throughput abattoir slaughters above 21 animals per day (MSA, 2000).

### 2.3 Study animals and design

**Fig. 2:**
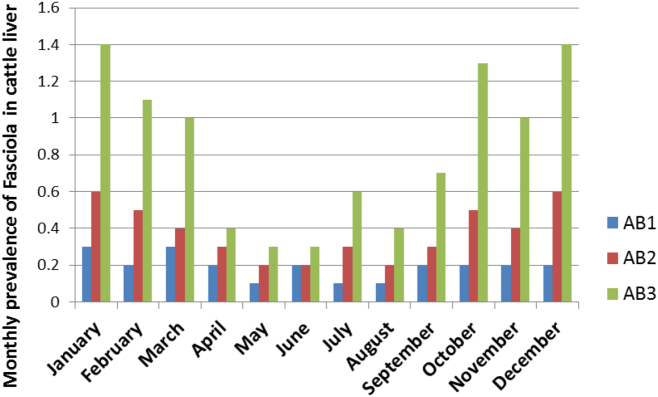
The summarized monthly prevalence of *Fasciola* in the liver of slaughtered cattle during the year 2010–2012 in two high throughput abattoirs (AB1 and AB2) and a low throughput abattoir (AB3).

The retrospective study of 78,728 cattle (AB1 = 62, 420, AB2 = 14, 719, AB3 = 1, 589) was extracted from abattoir records of 2010–2012 archived in the Department of Rural Development and Agrarian Reform (DRDAR). From these records, the number of liver condemned due to of *Fasciola* infection and the monetary loss was estimated. From same data, the annual and monthly prevalence of *Fasciola* was obtained for each abattoir (see Fig. 2).

The prospective study involving post-mortem meat inspection (PMMI) was carried out by International Meat Quality Assurance Service (IMQAS), qualified meat inspectors, who had undergone specialised training in meat inspection, processing, disease identification and pathology of farm animals. All animals included in this study were brought to the abattoir from nearby municipalities and districts. The inspectors carried out their work under occasional supervision by state veterinarians and officials of the Directorate of Veterinary public health. The meat inspectors routinely incise and visually inspect organs from slaughtered cattle for gross pathology. Infected livers were declared unfit for human consumption, hence are condemned. All rejected organs were recorded in standard data sheet approved by the Veterinary Public Health Department. Condemned livers were counted and weighed before disposal. A typical case sampling technique was adopted for the survey, this sampling method allows for the convenient examination of all liver and the purposeful selection of all livers grossly infected with *Fasciola*. The post-mortem meat inspection (PMMI) was carried out by the procedures outlined by the meat safety act of 2000 (MSA, 2000).

### 2.4 Financial loss estimation associated with liver condemnation

The total economic loss for the retrospective years (2010 − 2012) was calculated using the mean number of liver examined in the year of study to the number of whole liver condemned in the same year. It was multiplied by the mean price for one liver (ZAR 55) and the prevalence of fasciolosis in each of the abattoir.

In the prospective study, the number of whole liver condemned was recorded as well as the number of the partially condemned liver. The financial loss was estimated by multiplying the number of the condemned liver, the price of the liver (ZAR 55), the mean number of liver examined during the PMMI and the prevalence of fasciolosis. A detailed formula for the financial loss was mathematically computed as shown in Table 1. The total annual economic loss due to the condemnation of liver was computed mathematically using the modified method set by Ogurinade and Ogunrinade (1980).

**Table 1:** The formula for estimating economic losses and explanation of abbreviation.

Formula	Explanation of abbreviation
AL_LC_ = MCS × MLC × P	AL_LC_: Annual loss from liver condemnation
	MCS_retro_: Mean number of cattle slaughtered per retrospective year at abattoirs (AB1, AB2 and AB3)
	MCS_pmmi_: Total number of cattle examined during PMMI at abattoirs (AB1, AB2 and AB3)
	MCL: Mean cost of one liver at the abattoirs
	P: Prevalence of *Fasciola* in cattle liver at the each abattoir
AL_PLC_ = MCS_pmmi_ × ½ MLC × P	AL_PLC_: Annual loss from partial liver condemnation
	MCS_pmmi_: Total number of cattle examined during PMMI at abattoirs (AB1, AB2 and AB3)
	½ MCL: Mean cost of half liver at the abattoirs
	P: Prevalence of *Fasciola* in cattle liver partially condemned at the abattoirs
TAEL = AL_LC_ + AL_PLC_	TAEL: Total annual economic loss of liver due to fasciola infection
	ZAR: South African Rand, USD: USA Dollars

(Ogurinade and Ogunrinade, 1980).

### 2.5 Data analysis

All data was captured into Excel (Microsoft corporation WA, USA), transformed and analyzed using Microsoft Excel mathematical functions. Furthermore, descriptive statistics were generated using SPSS (Statistical Package for Social Sciences version 22). The prevalence of fasciolosis was defined as the proportion of liver condemned to the total number of liver examined during the study.

## 3 Results

**Fig. 3:**
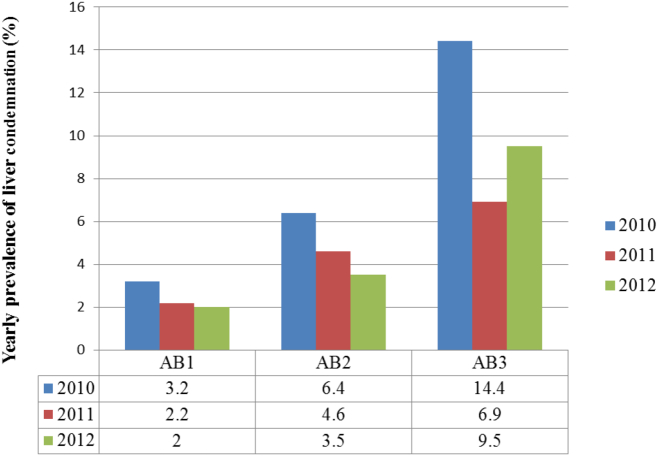
Percentage prevalence of liver condemned due to fasciolosis in slaughtered cattle from 2010 to 2012 in 3 abattoirs.

The result for the retrospective study as shown in Fig. 1, Fig. 2 indicates that prevalence of *Fasciola* was greater in the wet and warm season (October–March) than in cold and dry season (April–September). The overall result shows a gradual decline in condemnation of liver due to *Fasciola* infection at AB1 and AB2. But at AB3, there was an undulating increase in the prevalence of *Fasciola* over the three years studied. In the year 2010, 2011 and 2012, a total of 9774, 21803 and 30843 livers were examined, and 311, 484 and 611 livers were condemned due to *Fasciola* at AB1. The prevalence of *Fasciola* was 3.2, 2.2 and 2.0 in the respective study year. At AB2, a total of 4414, 4078 and 6227 livers were inspected in 2010, 2011 and 2012 and 281, 187 and 216 livers were condemned representing 6.4, 4.6 and 3.5% for the 2010–2012. While meat inspectors examined 437, 520 and 632 livers at the low throughput abattoir (AB3) during the same period and condemned 63, 36 and 60 livers. The prevalence of *Fasciola* infection at AB3 was14.4, 6.9 and 9.5% for 2010–2012 (Fig. 3). Total economic loss and losses associated with whole and partial liver condemnations are summarized in Table 2, Table 3. Briefly, in the retrospective period (2010–2012) financial loss due to whole liver condemnation was estimated as ZAR 129, 901 (9992.4 USD).

**Table 2:** Summarized (2010–2012) annual financial losses associated with whole liver condemnation and carcass weight loss due to fasciolosis in three abattoirs (*n* = 62,420, 14,719 and 1589) for AB1, AB2 and AB3 respectively.

Formula and abbreviation	Abattoirs			Total
	AB1	AB2	AB3	
zAL_LC_ = MCS_retro_ × MLC × P				
No condemned	1406	684	159	2249
MCS_retro_	62,420	14,719	1589	78,728
MLC (ZAR)	55	55	55	55
P (%)	0.02	0.05	0.1	0.03
ALC (ZAR)	68,662	40,477	8740	129,901
USD	5281.7	3113.6	672.3	9992.4

**Table 3:** Annual financial losses associated with whole and partial liver condemnation and carcass weight loss due to fasciolosis in three abattoirs during the study from July 2013 to June 2014.

Formula and abbreviation	Abattoirs			Total
	AB1	AB2	AB3	
AL_LC_ = MCS_pmmi_ × MCL × P				
No condemned	89	57	18	164
MCS_pmmi_	2570	519	53	3142
MLC (ZAR)	55	55	55	55
P (%)	0.178	0.143	0.082	0.146
ALC (ZAR)	1935.4	314.0	18.4	1940.8
USD	2, 351	381	22	2, 357

AL_PLC_ = MCS_pmmi_ × ½ MCL × P				
No trimmed	109	94	53	256
MCS	2570	519	53	3142
½ MCL (ZAR)	27.5	27.5	27.5	27.5
P (%)	0.218	0.235	0.241	0.228
ALC (ZAR)	15, 407	3, 354	351.3	19, 700
USD	1185.2	258.0	27.0	1515.4
TAEL (ZAR) = AL_LC_ + AL_PLC_	40,567	7436	590.3	44,930
USD	3120.5	572.0	45.4	3456.2

**Fig. 4:**
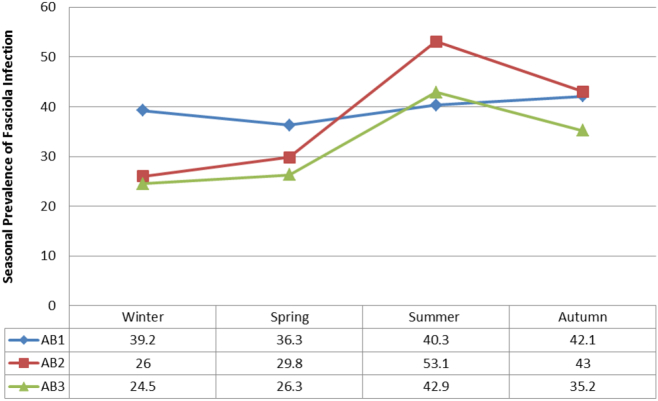
Percentage prevalence of liver condemnation due to fasciolosis from July 2013 to June 2014.

Seasonal prevalence of *Fasciola* infection during the prospective survey (July 2013 to June 2014) shows that more liver were condemned in the summer (AB1 = 40.3, AB2 = 53.1and AB3 = 42.9) than in winter (AB1 = 39.2, AB2 = 26 and AB3 = 24.5) (Fig. 4) (see also Table 2). The total economic loss ascribable to fasciolosis in cattle was ZAR 44, 930 (3456.2 USD). The financial loss recorded during this time was due to the condemnation of 164 whole livers and 256 partially trimmed livers (Table 3).

## 4 Discussion

The prevalence of fasciolosis in the retrospective study ranged from 2% to 14.4% while during the prospective study the prevalence was ranged from 24.5% to 53.1%. To the best of our knowledge reports on the prevalence and economic losses due to fasciolosis in South Africa are scanty (Kock et al., 2003, Mucheka et al., 2015). But *Fasciola* distribution in the Free State Province and DNA sequence analyses of novel haplotypes of *F. gigantica* with *F. hepatica* in KwaZulu Natal Province has been reported (Mucheka et al., 2015, Tsotetsi and Mbati, 2003).

Elsewhere, numerous surveys on the prevalence of fasciolosis in slaughtered animals have been reported and concurs with the findings in the present study (Asif Raza et al., 2007, Elliott et al., 2015, Khoramian et al., 2014, Mellau et al., 2011, Mwabonimana et al., 2010, Pfukenyi and Mukaratirwa, 2004, Phiri, 2006, Regassa et al., 2013, Usip et al., 2014, Wondwosen et al., 2012). The high prevalence recorded during the wet/warm season could be attributed to the activity of intermediate host *Galba truncatula* and Radix natalensis. Also, climate change could alter the transmission dynamics of *Fasciola* in endemic areas and enable it to transmit infection in regions where they were previously absent (Ashrafi and Mas-Coma, 2014, Fitzpatrick, 2013, Keiser and Utzinger, 2009, McLeod, 2011, Molina-Hernández et al., 2015, Novobilský et al., 2015, Torgerson and Macpherson, 2011).

The variation in the prevalence of *Fasciola* infection in AB1, AB2 and AB3 could be due to the abattoir sampling technique, slaughter capacity, meat inspection efficiency, adequate documentation and proper animal husbandry. AB3 is situated proximal to rural settlements and hence attract patronage by poor resourced smallholder farmer. These farmers lack modern farming techniques, access to veterinary assistance and extension services, as well as poor animal management techniques. Predisposing their livestock to disease-causing pathogens (Keyyu et al., 2006, Mapiye et al., 2009, Musemwa et al., 2008, Seimenis, 2012).

The financial loss of 2, 357 to 9992.4 USD due to *Fasciola* infection in this study portends grave danger to livestock production and sustainability. The consequence of bovine fasciolosis could be far-reaching and disastrous to animal health, South Africa's economy and food security.(Elliott et al., 2015, Greter et al., 2014, Van Wyk and Boomker, 2011).

Our study indicates that infection with *Fasciola* Spp. causes economic loss through liver condemnation. The impact of such loss can only be appreciated in the light of the high poverty index, hunger, and food insecurity in the Eastern Cape Province. The province is the second largest in terms of landmass, has the largest number of livestock and also high poverty index. The scarcity of animal health professionals such as Veterinary doctors signals a gap in primary animal health care system and failure in maintaining proper herd health programme. Hence, the endemicity of *Fasciola* infection in cattle may remain persistently high. A similar study in Turkey reported a relatively low estimate of 760 USD loss (Yibar et al., 2015), but a far greater loss of 85,051.70 USD, 2,567,586 USD and 5,110,499 USD was reported in Nigeria, Kenya, and Iran respectively (Cadmus and Adesokan, 2009, Jahed Khaniki et al., 2013, Kithuka et al., 2002). Aside from the economic losses described previously, the management and cost of treatment of animal infected with *Fasciola Spp*. is burdensome. Farmers spend on the average ZAR 15–30 per animal per treatment of *Fasciola* infection (Quayle et al., 2010). The paucity of veterinary and allied professional encourages the indiscriminate application of flukicidal regimen which has the potential to promote drug resistance.

The differences in the loss reported in this study and others may be attributed to different abattoir slaughter capacity, estimation methodologies, sampling techniques, livestock populations. Other reasons may be variations in prevalence, climatic conditions, and the productivity of animals and prices (Abunna et al., 2010, Alembrhan and Haylegebriel, 2013, Fekadu et al., 2012, Yibar et al., 2015). The prevalence and financial losses in the Province due to fasciolosis could have been under-reported since a significant proportion of cattle is slaughtered informally. Moreso, animals slaughtered for traditional and religious purposes rarely get inspected. Fasciolosis is further made worse by poor animal husbandry, lack of veterinary assistance, poverty, and ignorance of parasite prevalence and infection cycle (Musemwa et al., 2008, Ndou et al., 2011).The prevalence of *Fasciola* reported in the current study also portends great danger for public health. Hence, its potential as a re-emerging zoonosis should not be overlooked. However,

## 5 Conclusion

The prevalence of *Fasciola* infection in this study can be used as a baseline for future extensive epidemiological investigations in the country. Knowledge of the prevalence and losses due to fasciolosis creates an awareness of the significance of the parasites and their public health importance as zoonoses. Condemnation of the liver of infected cattle caused a significant financial loss as well as the loss protein food source. This loss, if not eliminated through effective control strategies, would have a major impact on the sustainability of the livestock industry. The strict and proper herd health program at the farm level and an intensified primary animal health programmes to eliminate parasite burden in animals should be initiated. Good slaughter practices coupled with adequate monitoring and training of meat inspectors will aid in the proper documentation of disease conditions at the abattoir. Further studies are necessary to determine the prevalence of *Fasciola* species infecting animals in the Province and the identity of the intermediate host and the risk factors of the diseases.

## 6 Limitations of the study

The present study was conducted only in three of the 90 red meat abattoirs. The province is relatively large, and with limited resources, the researchers could not visit all abattoir for sampling. The other limitation would be that of the low sensitivity of traditional visual only meat inspection performed by the meat inspector; this cannot detect acute and sub-acute *Fasciola* infection. Finally, there was no data regarding partial liver condemnation in the retrospective data (2010–2012). Hence it is very likely that the true prevalence and financial loss due to fasciolosis are considerably higher than the reported prevalence.

## Competing interests

The authors declare that they have no competing interests.
